# Supplementary material for: Collagen 1A1 (COL1A1) Is a Reliable Biomarker and Putative Therapeutic Target for Hepatocellular Carcinogenesis and Metastasis
Source: Cancers (Basel). 2019 Jun 7;11(6):786. doi: 10.3390/cancers11060786 (PMC6627889; doi:10.3390/cancers11060786)
Supplement: Supplementary file 1 [file cancers-11-00786-s001.pdf]

Supplementary Materials: Collagen 1A1 (COL1A1) Is a Reliable Biomarker and Putative Therapeutic Target for Hepatocellular Carcinogenesis and Metastasis

Hon-Ping Ma, Hang-Lung Chang, Oluwaseun Adebayo Bamodu, Vijesh Kumar Yadav, Ting-Yi Huang, Alexander T. H. Wu, Chi-Tai Yeh, Shin-Han Tsai and Wei-Hwa Lee

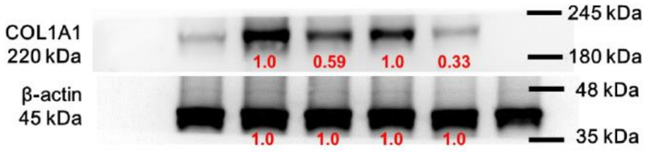

Figure S1. Full-size blots of Figure 3A.

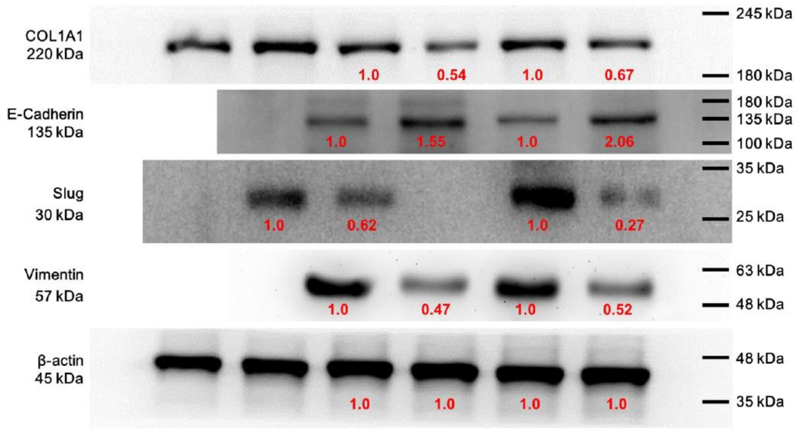

Figure S2. Full-size blots of Figure 3D.

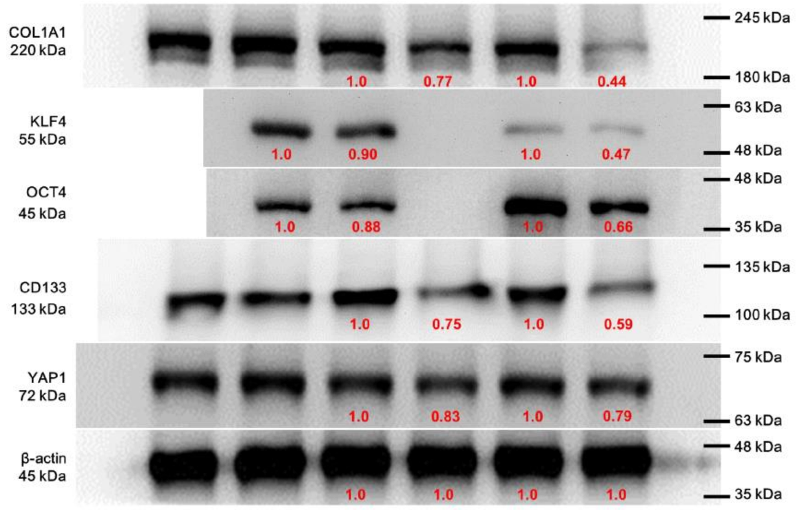

Figure S3. Full-size blots of Figure 5C.

Table S1. Western blot antibodies sheet.

No.	Target	Dilution	Catalog	kDa
1	COL1A1	1:1000	COL1A1 Antibody #84336	220
2	β-actin	1:10,000	β-Actin (8H10D10) Mouse mAb #3700	45
3	E-Cadherin	1:1000	E-Cadherin (24E10) Rabbit mAb #3195	135
4	Slug	1:1000	Slug (C19G7) Rabbit mAb #9585	30
5	Vimentin	1:1000	Anti-Vimentin antibody (ab137321)	57
6	KLF4	1:1000	Anti-KLF4 antibody (ab129473)	55
7	OCT4	1:1000	Oct-4 Antibody #2750	45
8	CD133	1:1000	Anti-CD133 antibody (ab19898)	133
9	YAP1	1:1000	Anti-YAP1 antibody (ab56701)	72

© 2019 by the authors. Licensee MDPI, Basel, Switzerland. This article is an open access article distributed under the terms and conditions of the Creative Commons Attribution (CC BY) license (http://creativecommons.org/licenses/by/4.0/).
